# Self-Care Theory and Translation to Intervention

**DOI:** 10.1093/geroni/igab046.1452

**Published:** 2021-12-17

**Authors:** Karen Hirschman, Barbara Riegel

**Affiliations:** University of Pennsylvania, Philadelphia, Pennsylvania, United States

## Abstract

Self-care is defined as a process of maintaining health through health promoting practices and managing illness when it occurs. Self-care is integral in the management of chronic conditions, but even those without illness engage in some level of self-care daily. In our on-going study we promote self-care as a means to control the stress associated with caregiving. We acknowledge the burden of caregiving for a loved one experiencing a serious chronic illness. That responsibility is typically associated with significant stress for the caregiver. We use stress theory to address the caregivers’ appraisal of events and coping responses. Three experienced health coaches were hired to provide 10 sessions of coaching over a 6-month period to each of the caregivers randomized to the intervention group. The emphasis of the iCare4Me coaching sessions is to address primary and secondary appraisal and coping as a means to improve self-care and thereby decrease stress.

